# Effect of a mesoporous NiCo_2_O_4_ urchin-like structure catalyzed with a surface oxidized LiBH_4_ system for reversible hydrogen storage applications†

**DOI:** 10.1039/d4ra01709a

**Published:** 2024-07-02

**Authors:** Ajaijawahar Kaliyaperumal, Gokuladeepan Periyasamy, Iyakutti Kombiah, Karthigeyan Annamalai

**Affiliations:** a Hydrogen Storage Materials and Nanosensors Laboratory, Department of Physics and Nanotechnology, College of Engineering and Technology, SRM Institute of Science and Technology Kattankulathur Chengalpattu Tamil Nadu India 603203 karthiga@srmist.edu.in +91 9841615368

## Abstract

A mesoporous NiCo_2_O_4_ urchin-like structure was synthesized by applying a facile hydrothermal method. Different concentrations of NiCo_2_O_4_ urchin-like structures were mixed with a surface oxidized LiBH_4_ system using a wet-impregnation method, followed by heat treatment. The hydrogen storage capacity of LiBH_4_ + 25% NiCo_2_O_4_, LiBH_4_ + 50% NiCo_2_O_4_ and LiBH_4_ + 75% NiCo_2_O_4_ systems was investigated. Typically, hydrogenated LiBH_4_ + 25% NiCo_2_O_4_, LiBH_4_ + 50% NiCo_2_O_4_ and LiBH_4_ + 75% NiCo_2_O_4_ systems desorbed 2.85 wt%, 3.78 wt% and 3.91 wt% of hydrogen, respectively, at the dehydrogenation temperature ranging from room temperature (RT) to 275 °C. Further, the LiBH_4_ + 75% NiCo_2_O_4_ system exhibited better kinetics than other systems and released ∼5.8 wt% of hydrogen at a isothermal dehydrogenation temperature of 250 °C in 60 minutes. Hydrogen binding energies were calculated as 0.28 eV, 0.27 eV and 0.26 eV for LiBH_4_ + 25% NiCo_2_O_4_, LiBH_4_ + 50% NiCo_2_O_4_ and LiBH_4_ + 75% NiCo_2_O_4_ systems, respectively. Moreover, the calculated activation energies of LiBH_4_ + 25% NiCo_2_O_4_, LiBH_4_ + 50% NiCo_2_O_4_ and LiBH_4_ + 75% NiCo_2_O_4_ systems are 17.99 kJ mol^−1^, 17.03 kJ mol^−1^ and 16.92 kJ mol^−1^, respectively. The calculated BET (Brunauer–Emmett–Teller) surface area of NiCo_2_O_4_ and LiBH_4_ + 75% NiCo_2_O_4_ systems is 124.05 and 136.62 m^2^ g^−1^, respectively. These results showed that hydrogen sorption and desorption properties are significantly increased by the influence of mesoporous structure, lower binding energy and activation energy of LiBH_4_ + 75% NiCo_2_O_4_ system.

## Abstract 2

Surface oxidized LiBH_4_/NiCo_2_O_4_ systems prepared by wet-impregnation method. LiBH_4_ + 75% NiCo_2_O_4_ released 5.8 wt% H_2_ at 250 °C in 60 min. The increased concentration of NiCo_2_O_4_ in all the systems impacts the active sites and H_2_ storage capacity.

## Graphical abstract

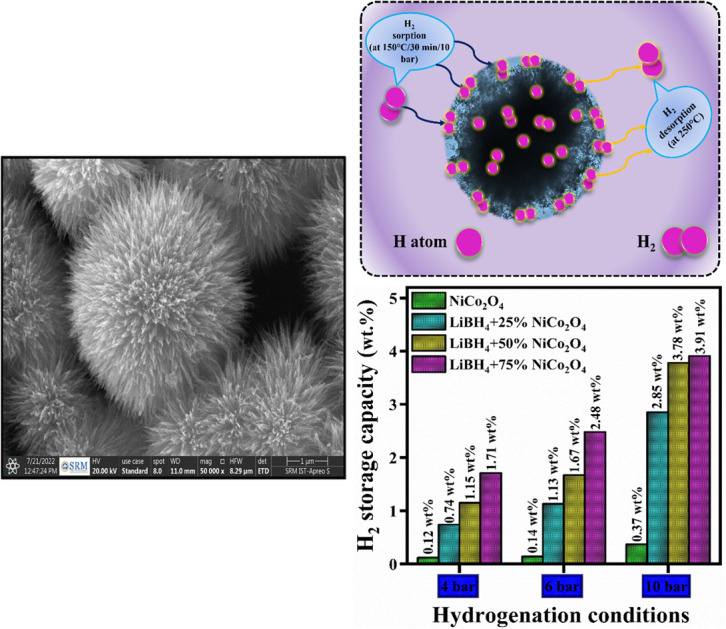

## 1 Introduction

In the current scenario, over 80% of the global energy supply is obtained from conventional non-renewable fossil fuels, including natural gas, coal and petroleum sources.^1,2^ The use of fossil fuels releases greenhouse gases and other polluting substances that seriously harm the environment.^3^ Hydrogen has been recognized as a abundant, clean, economical and efficient energy source for a wide range of applications, including stationary supplies, distribution and various transportable hydrogen-fuelled systems. Hydrogen is an excellent long-term option to overcome the energy-based environmental issues, due to its advantages of high energy density (120 MJ kg^−1^). During the energy conversion process, it just produces water as an output product.^4–7^ However, the primary challenge among them is the absence of suitable techniques for storing the hydrogen. Moreover, storing hydrogen using traditional methods, such as compression and liquefaction, requires tremendous pressure and/or very low temperatures, which in turn increases the chances of leakage and safety issues.^8,9^

Solid-state hydrogen storage is a feasible technique to achieve future hydrogen storage goals considering its safety conditions, compactness and efficiency compared to conventional hydrogen storage systems.^10,11^ Hydrogen can be bound either physically or chemically with different storage materials, which has a definite advantages in the aspects of safety and efficiency.^12,13^ In recent decades, LiBH_4_ has been widely explored^14,15^ as a reversible hydrogen storage material for on-board energy carrier applications owing to its high volumetric and gravimetric hydrogen densities. Its slow kinetics, higher dehydrogenation temperature and severe reversible conditions (600 °C/35 MPa H_2_) are major concerns. This behaviour can be associated with its high thermal stability and poor catalytic activity.^16^ The complex hydrides LiBH_4_,^17–21^ NaBH_4_,^22,23^ and LiH^24^ are extremely sensitive to air/moisture in the ambient atmosphere. These materials react instantly with water through exothermic reactions. Therefore, the certain operating conditions such as dry air and inert atmosphere are required to prevent the dehydrogenation of raw materials. Consequently, it is essential to understand how raw materials behave under moisture conditions. To manage the reactivity and unfavourable impacts at each stage of the industrial process, including storage, manufacturing, handling and processing, many possible reaction strategies are suggested for handling LiBH_4_ under ambient atmospheric conditions according to the report by Goudon *et al.*^17^ However, for on-board applications, the reversibility of LiBH_4_ is still challenging.^25,26^

Recently, transition metal oxides have been used to improve the reversible hydrogen storage performance of complex hydrides through nanoconfinement and nanocatalytic effects. Some experimental studies involving LiBH_4_ with transition metal oxides have been reported for hydrogen storage applications.^27–29^ Zhang and his co-workers^30^ reported that the re-hydrogenated (at 400 °C under 4.5 MPa) LiBH_4_/M-Fe_2_O_3_/TiF_3_ system absorbed ∼4.27 wt% of hydrogen within 60 minutes. In a previous study, ∼1.7 wt% hydrogen uptake capacity for LiBH_4_–2LiNH_2_-0.05/3Co_3_O_4_ systems was found under rehydrogenation conditions of 25–220 °C and 110 bar H_2_ pressure.^31^ Additionally, the dehydrogenated LiBH_4_/SiO_2_/TiF_3_ system reabsorbed ∼1.2 wt% of hydrogen at 300 °C within 233 minutes under 4.5 MPa.^32^ Au *et al.*^33^ investigated the reversibility of LiBH_4_/TiO_2_ and LiBH_4_/V_2_O_3_ systems at 600 °C under 100 bar H_2_ pressure with the hydrogen uptake capacities of 7.8 wt% and 7.9 wt%, respectively. Zang and his colleagues^34^ reported the destabilization of the LiBH_4_ system by NiCo_2_O_4_ nanorods. The mesoporous NiCo_2_O_4_ nanorods were mixed with LiBH_4_ in different mass ratios (2 : 1, 1 : 1, 1 : 2) using the ball milling technique in a controlled atmosphere. As shown in their report, they directly dehydrogenated the raw LiBH_4_ system using the NiCo_2_O_4_ structure and did not hydrogenate and rehydrogenate the LiBH_4_–NiCo_2_O_4_ composites. Additionally, the *in situ* Co (Ni) and Co–B (Ni–B) are formed as intermediates during dehydrogenation. In this study, we used the surface oxidized LiBH_4_ system and mixed it with mesoporous NiCo_2_O_4_ urchin-like structure in different weight percentages (LiBH_4_ + 25% NiCo_2_O_4_, LiBH_4_ + 50% NiCo_2_O_4_ and LiBH_4_ + 75% NiCo_2_O_4_) by applying a simple wet-impregnation technique under lab atmospheric conditions. Moreover, we studied hydrogenation and dehydrogenation experiments systematically. From the hydrogenation and dehydrogenation results, it was found that no new phases were introduced into the LiBH_4_/NiCo_2_O_4_ systems. Cabo *et al.* and Zhang *et al.* found that Ni and Co are good candidates for hydrogen storage due to their variable valence and higher Pauling's electronegativity than Mn, Fe, Ti and other metals. It is reasonable to infer that NiCo_2_O_4_, Co_3_O_4_ and NiO have a notable influence on improving the hydrogen sorption/desorption properties of the MgH_2_ and LiBH_4_ systems. The aforementioned reports mostly present the destabilization effect of the LiBH_4_ system with sufficient hydrogen release at high temperatures.^28,31,35^ However, they have not been performed effectively in the reversible hydrogen storage process. Furthermore, nanostructured metal oxides present numerous advantages due to the hierarchical arrangement of pore structures with sizes ranging from nanometres to micrometres and catalytic activity. Moreover, the favourable hydrogen release mechanism depends on the mesoporous materials with large surface areas owing to more active sites to assist the dissociation/recombination of H_2_ molecules.^36,37^ Butt *et al.* investigated the hydrogen storage properties of hierarchical ZnV_2_O_4_ (ref. 38) and Zn_2_V_2_O_7_ (ref. 39) nanostructures, which absorbed 1.76 and 1.21 wt% of H_2_ under 5 MPa at 200 °C, respectively.

Under these scenarios, the present study focuses on investigating the hydrogen sorption/desorption performance of a mesoporous urchin-like NiCo_2_O_4_ structure catalyzed using a surface oxidized (air-sensitized) LiBH_4_ system. From our previously reported study of the hydrogen storage properties of a surface oxidized LiBH_4_ system catalyzed with NiO nanoflower,^19^ we adopted nanorods and nanoplates.^40^ Herein, the NiCo_2_O_4_ structure was prepared using the hydrothermal method, and LiBH_4_/NiCo_2_O_4_ systems were prepared using the ultrasonic-assisted wet-impregnation method. From scanning electron microscopy (SEM) and transmission electron microscopy (TEM) assessments, the mesoporous NiCo_2_O_4_ structure was found to have an urchin-like formation. According to Kang *et al.*,^41^ the surface oxidized LiBH_4_ could reduce the dehydrogenation and activation energy barrier during hydrogen desorption. In addition, the surface oxidation effects help to reach thermodynamically predicted temperatures for dehydrogenation reactions.^40,42,43^ In this study, it is observed that mesoporous NiCo_2_O_4_ with a surface oxidized LiBH_4_ system presents an outstanding storage performance. Notably, 5.8 wt% of hydrogen was desorbed using a LiBH_4_ + 75% NiCo_2_O_4_ system in isothermal dehydrogenation at 250 °C for 60 minutes. Moreover, the weak chemical binding nature and activation energy of the LiBH_4_/NiCo_2_O_4_ systems are discussed based on thermogravimetric analysis. Additionally, possible reaction mechanisms are discussed.

## 2 Experimental section

### 2.1 Materials

The commercial chemical LiBH_4_ was purchased from Sigma-Aldrich with ≥95.0% purity. Additional chemicals Ni(NO_3_)_2_·6H_2_O, Co(NO_3_)_2_·6H_2_O, CO(NH_2_)_2_, diethyl-ether (DEE) and ethanol were purchased from Siscon Research Laboratory, India, and used without further purification.

### 2.2 Methods

#### 2.2.1 Synthesis of nickel cobalt oxide

**Fig. 1:**
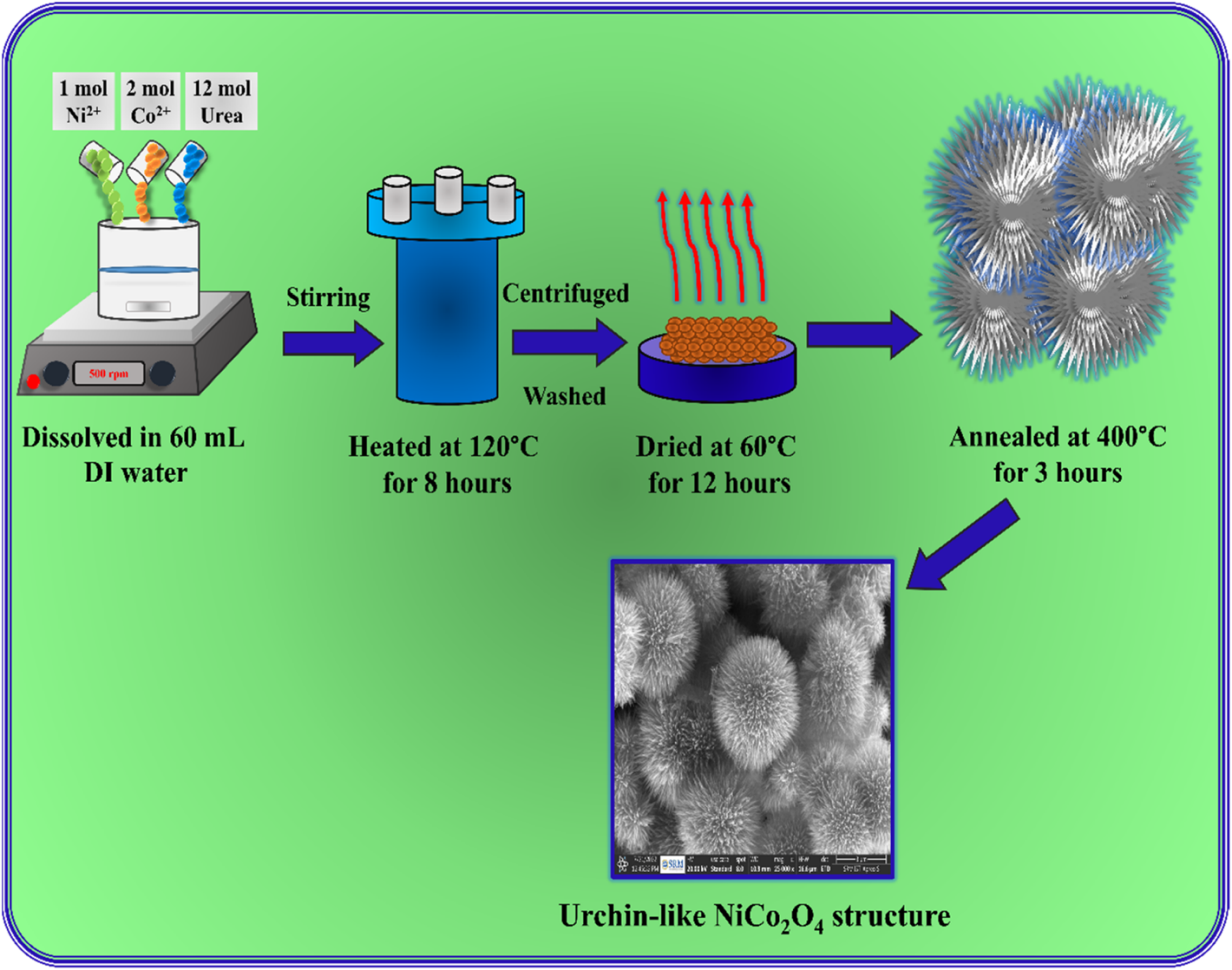
Schematic representation of the preparation of an urchin-like NiCo_2_O_4_ structure.

In a typical hydrothermal process, 1 mol Ni(NO_3_)_2_·6H_2_O and 2 mol Co(NO_3_)_2_·6H_2_O were dissolved in 60 mL distilled water, and 12 mol urea was added into the solution under continuous stirring for 60 minutes. Then, the solution was transferred into a 100 mL Teflon-lined stainless-steel autoclave and heated at 120 °C for 8 hours. After cooling to room temperature, the precipitate was centrifuged and washed several times with water and ethanol. Then, the precipitate was dried at 60 °C for 12 hours. Finally, the NiCo_2_O_4_ was obtained after annealing at 400 °C for 3 hours in air. Fig. 1 shows a schematic representation image of the urchin-like NiCo_2_O_4_ structure preparation.

#### 2.2.2 Synthesis of LiBH_4_/NiCo_2_O_4_ systems

LiBH_4_/NiCo_2_O_4_ systems were prepared using the wet-chemical impregnation method under ambient atmospheric conditions. Surface oxidized LiBH_4_ + 25% NiCo_2_O_4_ was dispersed in 30 mL of diethyl ether (DEE). The mixed solution was sonicated in a bath sonicator for 2 hours. Then, the resultant mixture was dried at 100 °C using a hotplate with vigorous stirring for solvent evaporation. The LiBH_4_ + 50% NiCo_2_O_4_ and LiBH_4_ + 75% NiCo_2_O_4_ were also prepared by applying the aforementioned procedure. Later, LiBH_4_ + 25% NiCo_2_O_4_, LiBH_4_ + 50% NiCo_2_O_4_ and LiBH_4_ + 75% NiCo_2_O_4_ mixtures were annealed at 275 °C for 1 hour under argon atmosphere.

### 2.3 Hydrogen sorption and desorption experiments

Before hydrogenation, the thermal stability of LiBH_4_/NiCo_2_O_4_ systems was confirmed by applying the TG-STA under an argon atmosphere. Then, 10–20 mg of the sample was placed in the hydrogenation chamber of the custom-built hydrogenation setup. Next, the sample was hydrogenated at 150 °C for 30 minutes under 4, 6 and 10 bar H_2_ pressure conditions. Immediately, the hydrogenated sample was transferred to the TG-STA for dehydrogenation from RT to 275 °C with a 15 °C min^−1^ rate under an argon gas flow of 100 mL min^−1^. Additionally, isothermal dehydrogenation experiments were performed at 250 °C for 1 hour using argon medium with 100 mL min^−1^ flow for the hydrogenated samples (at 150 °C for 30 minutes under 10 bar H_2_ pressure).

## 3 Results and discussion

### 3.1 Structural and functional group analyses

**Fig. 2:**
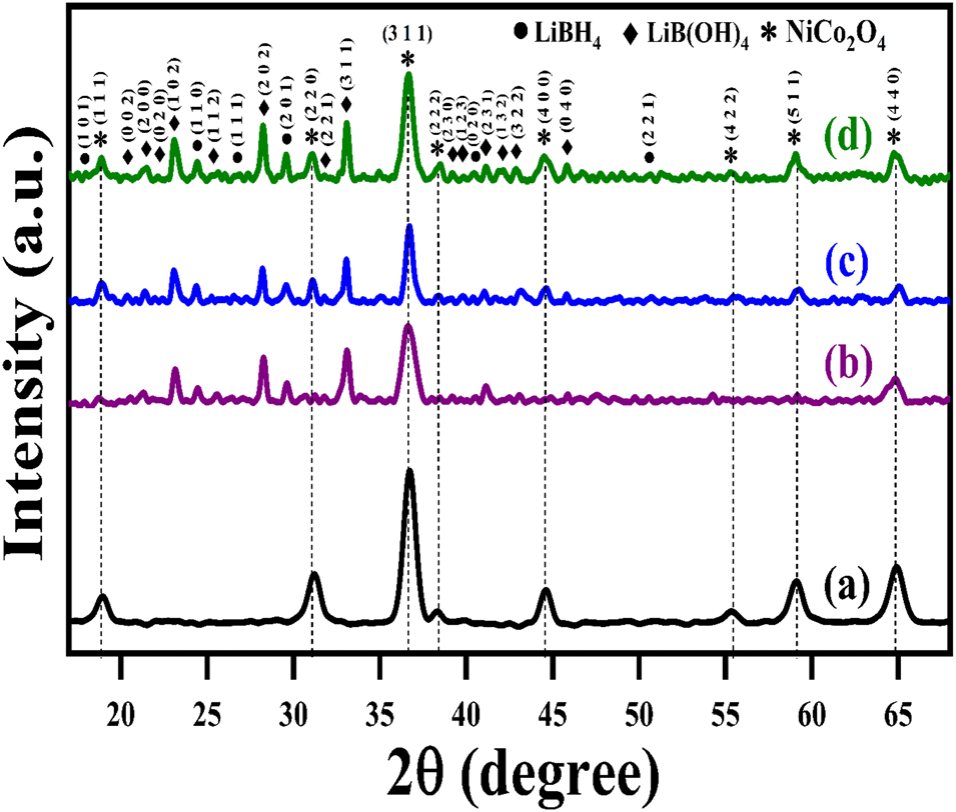
XRD patterns of (a) NiCo_2_O_4_, (b) LiBH_4_ + 25% NiCo_2_O_4_, (c) LiBH_4_ + 50% NiCo_2_O_4_, and (d) LiBH_4_ + 75% NiCo_2_O_4_ systems.

The structural analysis of NiCo_2_O_4_ and LiBH_4_/NiCo_2_O_4_ samples was performed using the powder X-ray diffraction (PXRD) technique. As shown in Fig. 2a, the diffracted peaks of NiCo_2_O_4_ indexed at 18.7°, 31.2°, 36.6°, 38.3°, 44.5°, 55.3°, 59.3° and 65.2°, corresponding to the (1 1 1), (2 2 0), (3 1 1), (2 2 2), (4 0 0), (4 2 2), (5 1 1) and (4 4 0) planes, respectively, are matched with standard data of JCPDS card no. #20-0781. The calculated average crystallite size is ∼26 nm. Further, the lattice constant values are found to be *a* = *b* = *c* = 8.11 Å for the NiCo_2_O_4_ structure. Fig. 2b–d shows that the XRD spectra of LiBH_4_ + 25% NiCo_2_O_4_, LiBH_4_ + 50% NiCo_2_O_4_ and LiBH_4_ + 75% NiCo_2_O_4_ systems indicate the presence of LiBH_4_ (JCPDS card no. #27-0287), LiB[OH]_4_ (JCPDS card no. 01-075-1156) and NiCo_2_O_4_ (JCPDS card no. #20-0781) phases. According to the results, it is clear that increasing the concentration of NiCo_2_O_4_ in the system produced an increased peak intensity. Additionally, the combined phases of borohydroxide (B[OH]_4_^−^) and borohydride (BH_4_^−^) were identified in the systems due to the surface oxidation of LiBH_4_.

**Fig. 3:**
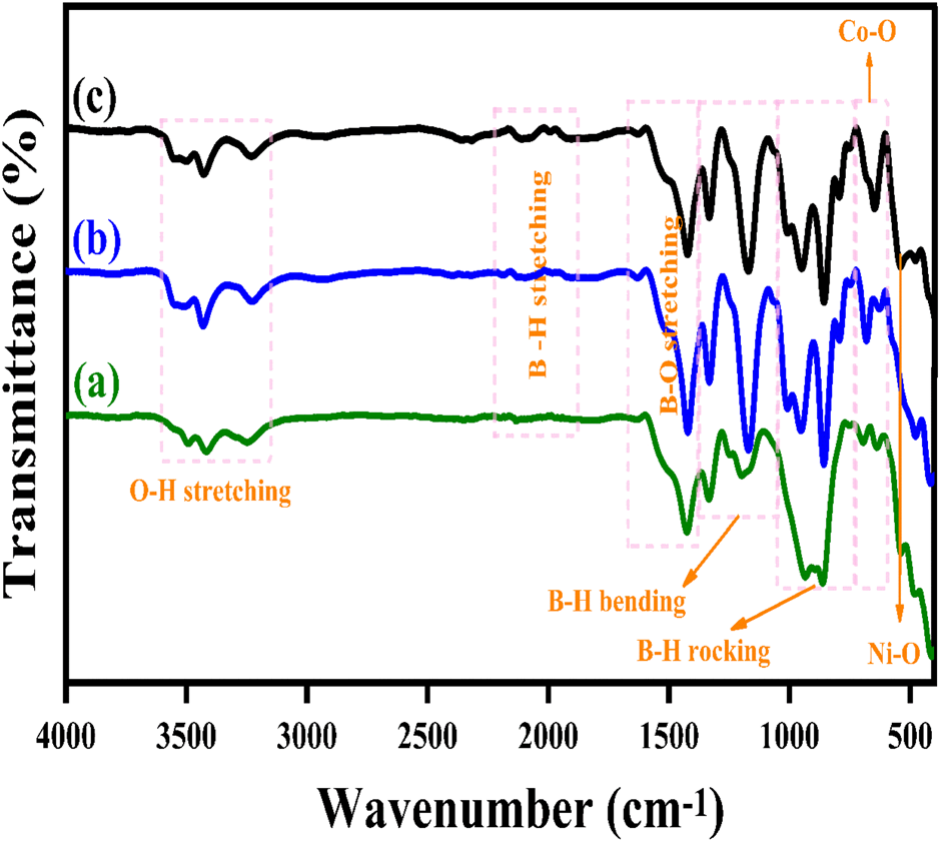
FTIR patterns of (a) LiBH_4_ + 75% NiCo_2_O_4_, (b) LiBH_4_ + 50% NiCo_2_O_4_,and (c) LiBH_4_ + 25% NiCo_2_O_4_ systems.

Fig. 3(a–c) illustrates the Fourier Transform Infrared Spectroscopy (FTIR) patterns of LiBH_4_ + 75% NiCo_2_O_4_, LiBH_4_ + 50% NiCo_2_O_4_, and LiBH_4_ + 25% NiCo_2_O_4_ systems. The characteristic bands of Ni–O and Co–O are observed at 551 and 645 cm^−1^,^44^ respectively, originating from the stretching vibration modes of the spinel NiCo_2_O_4_ structure. In addition, the vibration modes, such as O–H stretching (3592–3180 cm^−1^),^19^ B–H stretching (2484–2196 cm^−1^),^45^ B–O stretching (1559–1375 cm^−1^),^19^ B–H bending (1367–1007 cm^−1^)^45^ and B–H rocking (867–681 cm^−1^),^19^ can be observed in the spectra, confirming the presence of NiCo_2_O_4_, LiBH_4_ and LiB[OH]_4_. The air exposure of LiBH_4_ caused the existence of B[OH]_4_.

### 3.2 Hydrogen desorption analysis

**Fig. 4:**
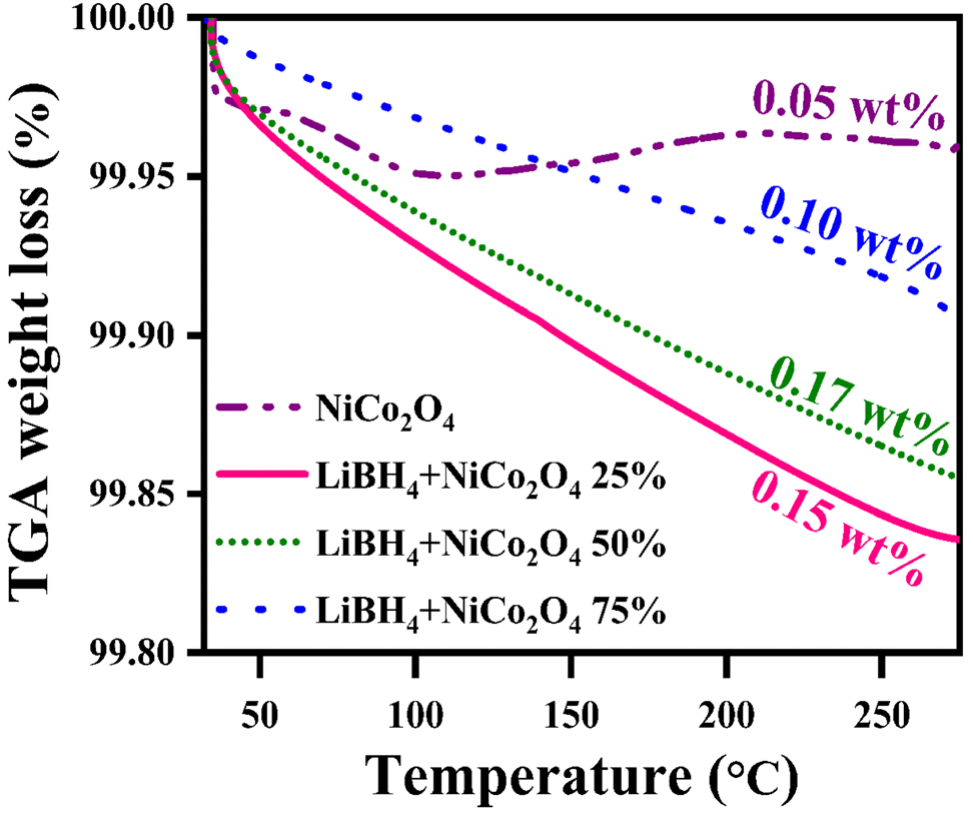
Thermal stability curves of NiCo_2_O_4_, LiBH_4_ + 25% NiCo_2_O_4_, LiBH_4_ + 50% NiCo_2_O_4_ and LiBH_4_ + 75% NiCo_2_O_4_ systems before hydrogenation.

**Fig. 5:**
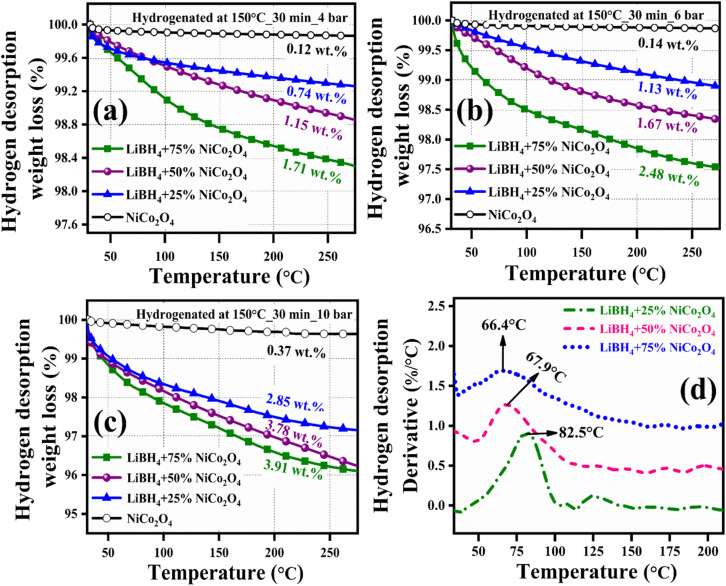
Non-isothermal hydrogen desorption curves of NiCo_2_O_4_, LiBH_4_ + 25% NiCo_2_O_4_, LiBH_4_ + 50% NiCo_2_O_4_ and LiBH_4_ + 75% NiCo_2_O_4_ systems: (a) Hydrogenated at 150 °C for 30 min under 4 bar, (b) hydrogenated at 150 °C for 30 min under 6 bar, and (c) hydrogenated at 150 °C for 30 min under 10 bar; (d) H_2_ desorption derivative TG curves of hydrogenated (150 °C for 30 min under 10 bar) LiBH_4_ + 25% NiCo_2_O_4_, LiBH_4_ + 50% NiCo_2_O_4_ and LiBH_4_ + 75% NiCo_2_O_4_ systems.

Before hydrogenation, the thermal stability of the systems was investigated using a thermal analyzer. As shown in Fig. 4, negligible 0.05, 0.17, 0.15 and 0.10 wt% of weight losses were observed in the systems, from RT to 275 °C. After confirming the thermal stability, all hydrogen sorption measurements were carried out at 150 °C for 30 minutes under 4 bar H_2_ pressure. The hydrogenated systems were dehydrogenated using a thermal analyzer from RT to 275 °C under an argon medium at a heating rate of 15 °C min^−1^. During non-isothermal hydrogen desorption, the hydrogenated NiCo_2_O_4_ system released 0.12 wt% of hydrogen, while the LiBH_4_ + 25% NiCo_2_O_4_, LiBH_4_ + 50% NiCo_2_O_4_ and LiBH_4_ + 75% NiCo_2_O_4_ systems gradually released 0.74, 1.15 and 1.71 wt% hydrogen, respectively, from RT to 275 °C in argon atmosphere (Fig. 5a). The hydrogen storage behaviour of the systems was analysed by increasing the hydrogenation conditions of 6 and 10 bar H_2_ pressures separately under a constant hydrogenation temperature of 150 °C for 30 minutes. As shown in Fig. 5b, the urchin-like NiCo_2_O_4_ structure liberated 0.14 and 0.37 wt% hydrogen from their adsorbed surface sites under the hydrogenation conditions of 6 and 10 bar, respectively. Meanwhile, LiBH_4_ + 25% NiCo_2_O_4_, LiBH_4_ + 50% NiCo_2_O_4_ and LiBH_4_ + 75% NiCo_2_O_4_ systems desorbed 1.13, 1.67 and 2.48 wt% of H_2_, respectively, for 6 bar pressure of hydrogenation. Moreover, under 10 bar hydrogenation condition, LiBH_4_ + 25% NiCo_2_O_4_, LiBH_4_ + 50% NiCo_2_O_4_ and LiBH_4_ + 75% NiCo_2_O_4_ systems released 2.85, 3.78 and 3.91 wt% of H_2_, respectively, from RT to 275 °C, as shown in Fig. 5c. From the above results, it can be inferred that the increased hydrogen sorption and desorption capacity of LiBH_4_/NiCo_2_O_4_ systems are attributed to the increased hydrogenation pressure.

To determine the binding and kinetics nature of the hydrogenated (150 °C for 30 minutes under 10 bar) LiBH_4_/NiCo_2_O_4_ systems, the activation and binding energies were calculated using Kissinger's^46–48^ and van't Hoff^47,48^ relations by analyzing the hydrogen desorption derivative thermogravimetric (DTG) curves. As shown in Fig. 5d, DTG curves exhibit the hydrogen desorption behaviour of LiBH_4_ + 25% NiCo_2_O_4_, LiBH_4_ + 50% NiCo_2_O_4_ and LiBH_4_ + 75% NiCo_2_O_4_ systems at the H_2_ desorption peak positions of 82.5, 67.9 and 66.4 °C, respectively. The corresponding activation energy (*E*_d_) and binding energy (*E*_b_) of the systems are, respectively, determined using the following equations:1
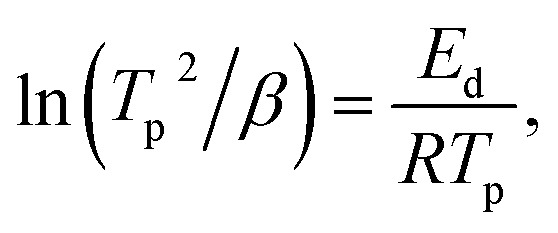
2
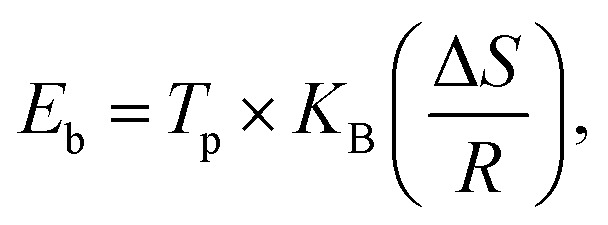
where *T*_p_ is the absolute temperature corresponding to the hydrogen desorption peak position, *β* is the heating rate, *E*_d_ is the activation energy, *E*_b_ is the ‘binding energy of 1H_2_ molecule, *K*_B_ is the Boltzmann's constant, Δ*S* is the change in entropy (75.44 J mol^−1^ K^−1^) and *R* is the ‘gas constant’. The calculated activation and binding energies of the systems are presented in Table 1.

**Table 1:** Hydrogen desorption characteristics of the systems under non-isothermal H_2_ desorption conditions

Systems	Desorption peak temperature	Desorption capacity up to 275 °C	Binding energy (*E*_b_)	Activation energy (*E*_d_)
LiBH_4_ + 25% NiCo_2_O_4_	82.5 °C	2.85 wt%	0.28 eV	17.99 kJ mol^−1^
LiBH_4_ + 50% NiCo_2_O_4_	67.9 °C	3.78 wt%	0.27 eV	17.03 kJ mol^−1^
LiBH_4_ + 75% NiCo_2_O_4_	66.4 °C	3.91 wt%	0.26 eV	16.92 kJ mol^−1^

For an ideal hydrogen storage material, the binding energy of hydrogen should fall in the range of ∼0.2–0.4 eV.^49–52^ The recommended binding energy range for physisorption is ∼0.01–0.1 eV and that for chemisorption is ∼2–3 eV. As shown in Table 1, the activation and binding energies of the systems lie in the ranges 16.92–17.99 kJ mol^−1^ and 0.26–0.28 eV, respectively. As suggested by Silambarasan *et al.* and Ioannatos *et al.*, this desorption activation energy is close to the adsorption energy in the case of strong physisorption and/or weak chemisorption, which is in the range of 10–40 kJ mol^−1^.^46,47^ According to previous studies, it is clear that the H_2_ is weakly chemisorbed on the LiBH_4_/NiCo_2_O_4_ networks.

Hydrogenation and dehydrogenation cycle studies were investigated for the selected LiBH_4_ + 75% NiCo_2_O_4_ system up to 5 times. Typically, hydrogenation experiments were carried out at 150 °C for 30 minutes under 10 bar pressure conditions for all hydrogenation cycles. Meanwhile, dehydrogenation cycle tests were conducted from RT to 275 °C under an argon atmosphere at a 15 °C min^−1^ heating rate. As shown in Fig. S1 (in ESI),† it was found to be ±0.25 wt% of variations in the H_2_ release by the system (3.76 wt%). Additionally, H_2_ sorption and desorption cycle tests confirm the stability of the systems, and further studies are required to evaluate their long-term performance.

**Fig. 6:**
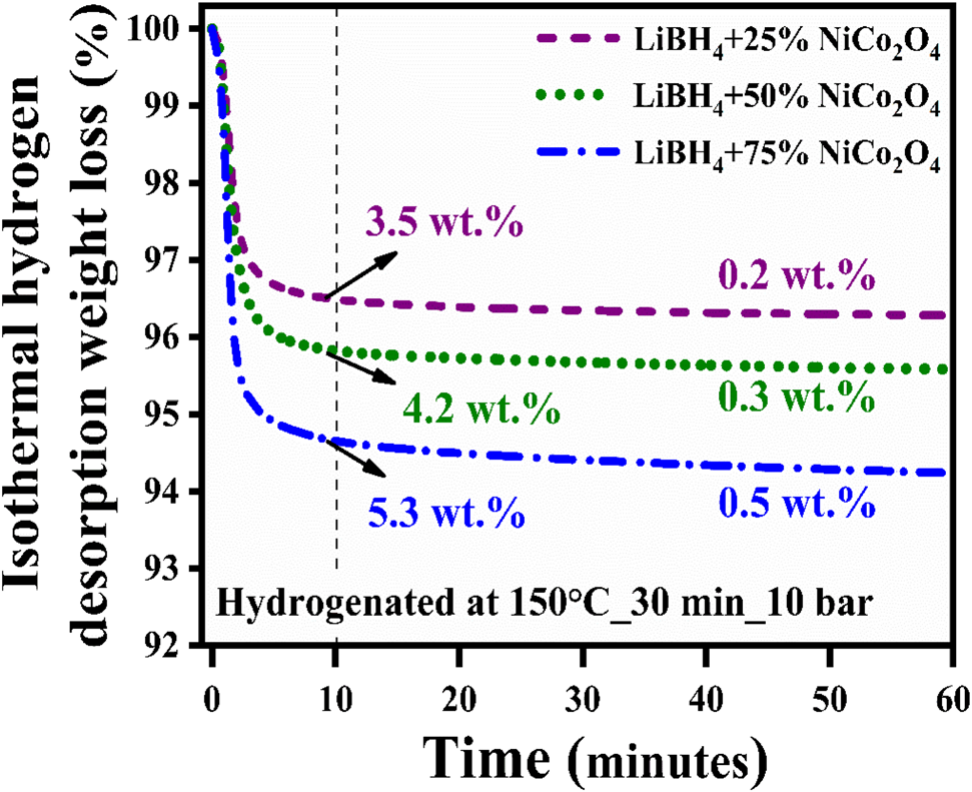
Isothermal hydrogen desorption curves of LiBH_4_ + 25% NiCo_2_O_4_, LiBH_4_ + 50% NiCo_2_O_4_ and LiBH_4_ + 75% NiCo_2_O_4_ systems.

Hydrogen desorption kinetics were studied under isothermal hydrogen desorption conditions at 250 °C for 60 minutes. Initially, the systems were hydrogenated at 150 °C for 30 minutes under 10 bar H_2_ pressure. As shown in Fig. 6, the LiBH_4_ + 75% NiCo_2_O_4_ system presented a higher desorption kinetics rate, and the capacity is 5.8 wt% in 60 minutes at 250 °C, while the LiBH_4_ + 25% NiCo_2_O_4_ and LiBH_4_ + 50% NiCo_2_O_4_ systems desorbed 3.7 and 4.5 wt% of hydrogen, respectively. Table 2 presents a comparison of the hydrogen desorption capacities of the LiBH_4_/NiCo_2_O_4_ systems in this study with those in other studies.

**Table 2:** Hydrogen storage properties of LiBH_4_ catalyzed with spinel metal oxide structures

System	Synthesis method	Isothermal hydrogen desorption capacity	Ref.
LiBH_4_ + 2LiNH_2_ + 0.05 wt% Co_3_O_4_	Ball milling at the argon-filled glove box	8.2 wt% (200 °C for 60 min)	53
LiBH_4_ + NiCo_2_O_4_		3.8 wt% (250 °C for 50 min)	34
LiBH_4_ + 9% mol NiFe_2_O_4_		2.4 wt% (300 °C for 20 min)	54
LiBH_4_ + 20 wt% M-Fe_2_O_3_ + 30 wt% TiF_3_		8.6 wt% (400 °C for 20 min)	55
LiBH_4_ + 2ZnO/ZnCo_2_O_4_	Wet chemical impregnation at argon	3.1 wt% (300 °C for 60 min)	56
LiBH_4_ + NiMnO_3_		2.8 wt% (300 °C for 60 min)	57
LiBH_4_ + 75% NiCo_2_O_4_^a^	Wet chemical impregnation in the air	5.8 wt% (250 °C for 60 min)	This work
LiBH_4_ + 50% NiCo_2_O_4_^a^		4.5 wt% (250 °C for 60 min)	
LiBH_4_ + 25% NiCo_2_O_4_^a^		3.7 wt% (250 °C for 60 min)	

a Hydrogen desorption condition after hydrogenation.

As shown in Table 2, the other systems also hold significant desorption kinetics through the destabilization effect. However, they did not report the rehydrogenation and then hydrogen desorption. Among other systems, the surface oxidized LiBH_4_/NiCo_2_O_4_ systems exhibit better hydrogen sorption kinetics.

### 3.3 Morphological and structural composition analyses

**Fig. 7:**
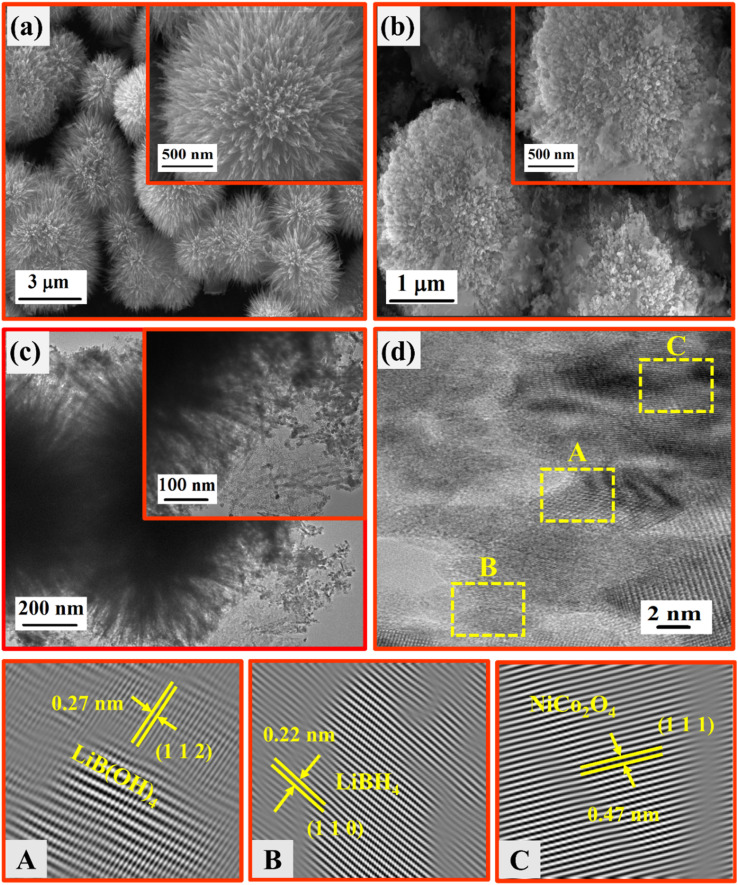
SEM images of (a) NiCo_2_O_4_ and (b) LiBH_4_ + 75% NiCo_2_O_4_; (c) TEM images of LiBH_4_ + 75% NiCo_2_O_4_; (d) HRTEM images of LiBH_4_ + 75% NiCo_2_O_4_.

SEM and TEM analyses were employed to investigate the morphological and structural nature of the NiCo_2_O_4_ and LiBH_4_ + 75% NiCo_2_O_4_ systems. As depicted in Fig. 7a, the SEM micrographs showed the urchin-like structure of NiCo_2_O_4_ with an average diameter of ∼4 µm, which consisted of several ultrafine nanoneedles. This nanoneedles-assembled urchin-like structure is attributed to the surfactant used in the reaction, causing more active sites at the nucleation stage.^58^ The SEM micrographs of the LiBH_4_ + 75% NiCo_2_O_4_ system are shown in Fig. 7b, exhibiting a molten form of LiBH_4_ with an urchin-like structure. TEM micrographs provide more insight into the morphology and structure of the LiBH_4_ + 75% NiCo_2_O_4_ system, as shown in Fig. 7(c) and (d). The average grain size is calculated as ∼19 nm, as shown in Fig. S2 (in ESI).† The interplanar *d*-spacing determined from HRTEM images (Fig. 7d) of LiBH_4_ + 75% NiCo_2_O_4_ system was 0.22, 0.27 and 0.47 nm,^59^ which were assigned to the lattice planes of (1 1 0), (1 1 2) and (1 1 1) for the LiBH_4_, LiB[OH]_4_ and NiCo_2_O_4_ phases, respectively. Fig. S3 (in ESI)† shows the selected area electron diffraction (SAED) pattern of the LiBH_4_ + 75% NiCo_2_O_4_ system, which showed that the polycrystalline nature and diffracted ring patterns agreed well with the XRD results.

### 3.4 Surface area analysis

**Fig. 8:**
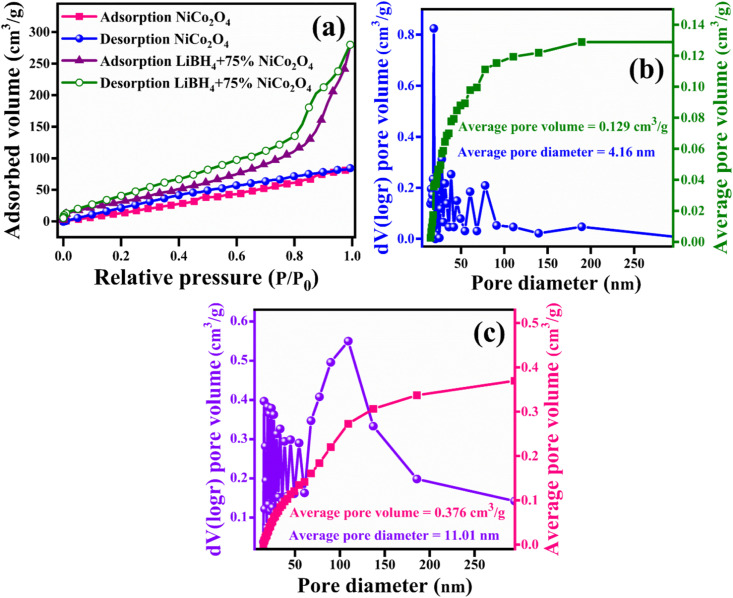
(a) Nitrogen adsorption/desorption curves of NiCo_2_O_4_ and LiBH_4_ + 75% NiCo_2_O_4_; BJH pore size distribution curves of (b) NiCo_2_O_4_ and (c) LiBH_4_ + 75% NiCo_2_O_4_.

For a better understanding of the H_2_ storage mechanism, BET analysis was carried out to measure the specific surface area and porous properties of NiCo_2_O_4_ and LiBH_4_ + 75% NiCo_2_O_4_ systems. As shown in Fig. 8a, the specific surface areas of NiCo_2_O_4_ and LiBH_4_ + 75% NiCo_2_O_4_ systems were determined by N_2_ adsorption/desorption isotherms. Both systems exhibit BET type IV isotherms. The BET surface areas for the NiCo_2_O_4_ and LiBH_4_ + 75% NiCo_2_O_4_ systems were 124.05 and 136.62 m^2^ g^−1^, respectively. As can be observed from the Barrett–Joyner–Halenda (BJH) pore size distribution curves, the NiCo_2_O_4_ (Fig. 8b) and LiBH_4_ + 75% NiCo_2_O_4_ (Fig. 8c) systems possess average pore volumes of 0.129 and 0.376 cm^3^ g^−1^; they exhibit average pore diameters of 4.16 and 11.01 nm, respectively. After the addition of NiCo_2_O_4_, the surface area and porous nature of the systems are slightly increased, contributing favourable H_2_ sorption and desorption to the LiBH_4_/NiCo_2_O_4_ systems.

### 3.5 Elemental and compositional analysis

**Fig. 9:**
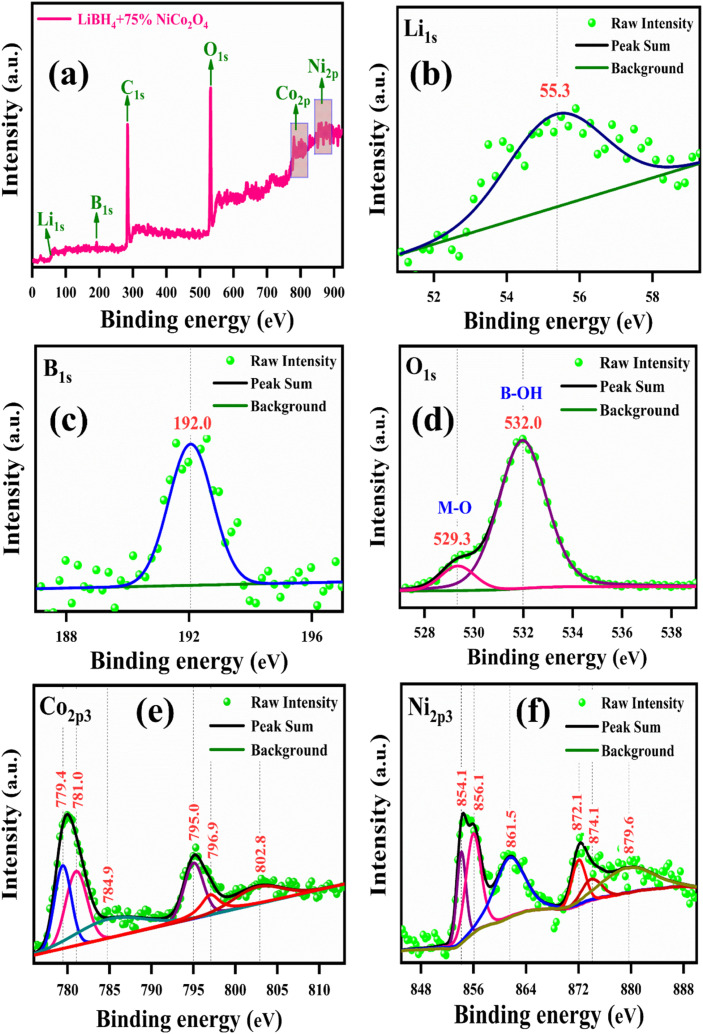
XPS spectrum of LiBH_4_ + 75% NiCo_2_O_4_: (a) survey spectrum, (b) Li 1s, (c) B 1s, (d) O 1s, (e) Co 2p_3_, and (f) Ni 2p_3_.

The binding energies and surface composition of the LiBH_4_ + 75% NiCo_2_O_4_ system were analyzed by applying the X-ray photoelectron spectroscopy (XPS) technique. The survey spectrum and high-resolution spectra of Li 1s, B 1s, O 1s, Ni 2p_3_ and Co 2p_3_ are presented in Fig. 9. The survey spectrum illustrates the presence of Li, B, O, Co and Ni elements (Fig. 9a). The binding energy of Li 1s is observed at 55.3 eV (ref. 18 and 60) (Fig. 9b), while B 1s is located at 192.0 eV (ref. 18 and 61) (Fig. 9c). Additionally, the peaks of O 1s are observed at the binding energies of 529.3 and 532.0 eV,^59^ which correspond to the typical metal–oxygen (M–O) and borohydroxide (B–OH) bonds, respectively (Fig. 9d). As shown in Fig. 9e, the high-resolution Co 2p_3_ spectrum is distributed into two Co species, that is the fitting peaks at 781.0 and 796.9 eV, which are attributed to the Co^2+^, and the peaks located at 779.4 and 795.0 eV are assigned to the Co^3+^ components. Additionally, satellite peaks are observed at 784.9 and 802.8 eV.^62^ Meanwhile, the Ni 2p_3_ spectrum is identified with Ni^2+^ and Ni^3+^ components having two satellite peaks. The deconvoluted peaks at 854.1 and 872.1 eV correspond to the Ni^2+^, while the peaks at 856.1 and 874.1 eV are due to the Ni^3+^. Moreover, the two satellite peaks are observed at binding energies of 861.5 and 879.6 eV (ref. 62) for Ni 2p_3_, as shown in Fig. 9f.

### 3.6 Structural analysis after hydrogenation, dehydrogenation and rehydrogenation

**Fig. 10:**
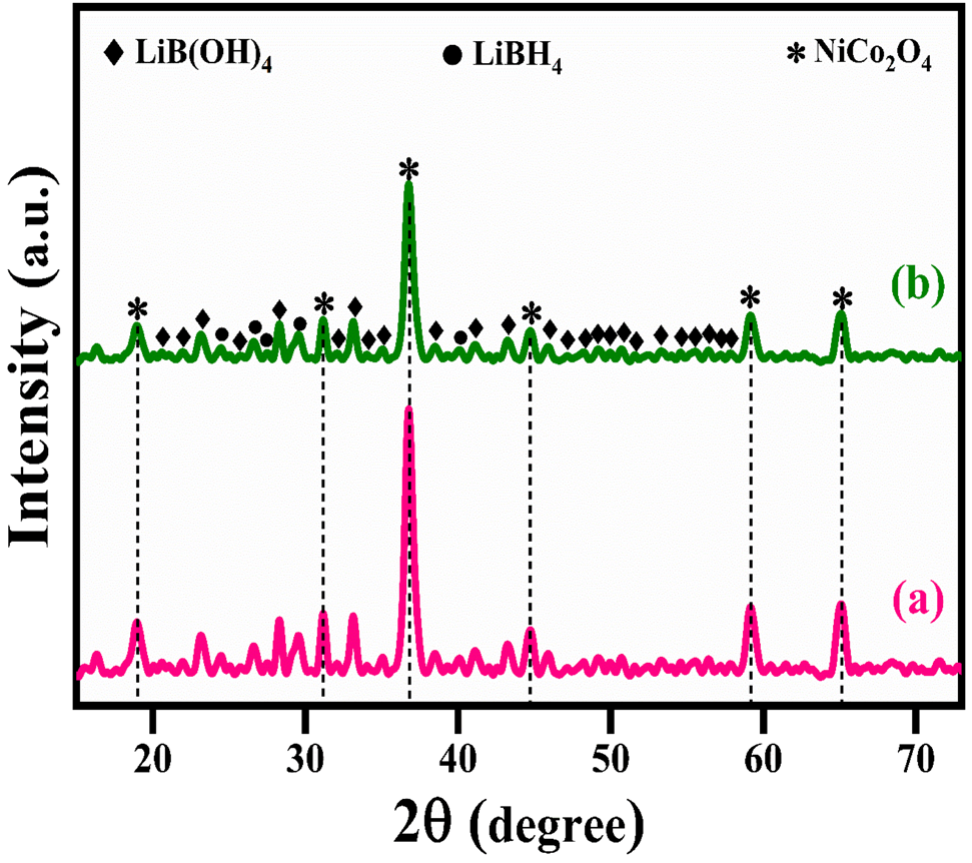
XRD patterns of LiBH_4_ + 75% NiCo_2_O_4_ (a) after hydrogenation (150 °C for 30 min under 10 bar) and (b) after dehydrogenation at 275 °C.

The microstructural changes in the LiBH_4_ + 75% NiCo_2_O_4_ system were characterized by XRD analysis after hydrogenation and dehydrogenation processes. Fig. 10(a) and (b) show the XRD pattern of the LiBH_4_ + 75% NiCo_2_O_4_ system after hydrogenation and dehydrogenation. Slight changes in peak intensities suggest the lattice rearrangement and defect refinement induced by the presence of hydrogen molecules.^57,63^ No structural changes were observed in the LiBH_4_ + 75% NiCo_2_O_4_ system after hydrogenation and dehydrogenation.^64^ The average crystallite size (*D*), micro-strain (*ε*) and dislocation density (*δ*)^65–69^ are presented in Table 3. From the structural analysis, it can be inferred that the hydrogen charging and discharging conditions did not alter the phase structures of the systems. Further theoretical and experimental investigations on these systems would lead to a better understanding of catalytic and synergistic mechanisms.

**Table 3:** Microstructural characteristics of the LiBH_4_ + 75% NiCo_2_O_4_ system

Process	Average crystallite size (nm)	Average dislocation density (×10^−3^ nm^−2^)	Average micro strain (×10^−3^)
Hydrogenated at 150 °C for 30 minutes under 10 bar	58	2.95	5.11
Dehydrogenated at 275 °C	55	3.15	5.49

**Fig. 11:**
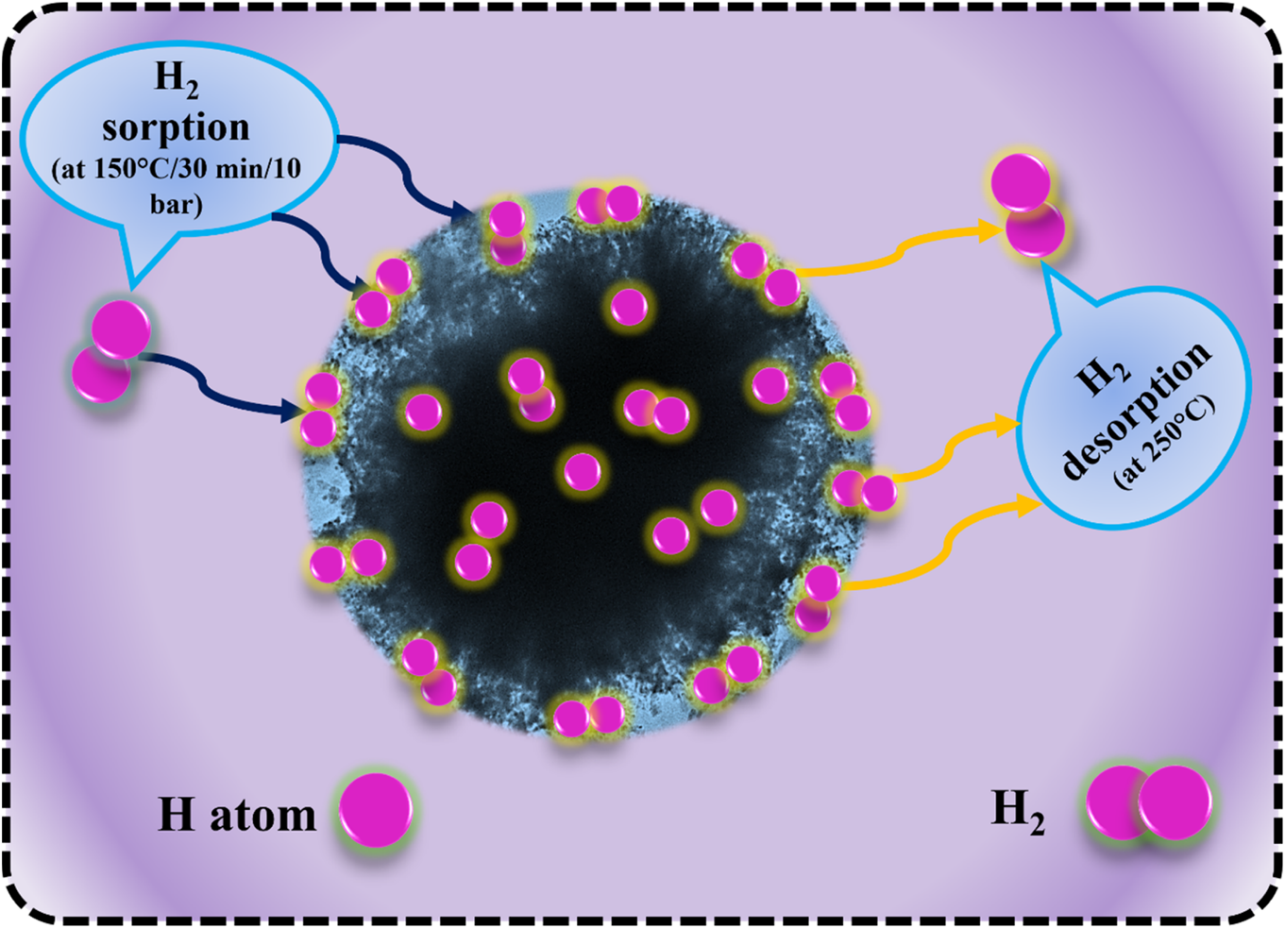
Hydrogen sorption and desorption mechanisms of LiBH_4_/NiCo_2_O_4_ systems.

Fig. 11 illustrates the schematic diagram of hydrogen sorption and desorption mechanisms for LiBH_4_/NiCo_2_O_4_ systems. During hydrogenation, hydrogen molecules diffused through the surface pores, reaching the interfaces of LiBH_4_/NiCo_2_O_4_ systems. It is expected that hydrogen can be absorbed in molecular and dissociated forms. In the following dehydrogenation process, the absorbed H_2_ molecules/hydrogen ions from the surfaces and interfaces of LiBH_4_/NiCo_2_O_4_ systems were desorbed at a dehydrogenation temperature of 275 °C. This suggests that the increased concentration of NiCo_2_O_4_ in the system impacts the active sites of LiBH_4_/NiCo_2_O_4_ systems and causes improved storage capacity.

## 4 Conclusion

The mesoporous NiCo_2_O_4_ was synthesized using the hydrothermal method, and a simple ultrasonication-assisted wet-impregnation method was used for the preparation of LiBH_4_/NiCo_2_O_4_ hydrogen storage systems. The physico-chemical properties were studied using XRD, FTIR, XPS, SEM, TEM, BET and TGA analyses. Furthermore, hydrogenation experiments were performed for the LiBH_4_ + NiCo_2_O_4_ systems at a hydrogenation temperature of 150 °C for 30 min under different pressures. From the non-isothermal dehydrogenation results, LiBH_4_ + 25% NiCo_2_O_4_, LiBH_4_ + 50% NiCo_2_O_4_ and LiBH_4_ + 75% NiCo_2_O_4_ systems exhibited the hydrogen release of 2.85, 3.78 and 3.91 wt%, respectively. Isothermal dehydrogenation of the hydrogenated LiBH_4_ + 75% NiCo_2_O_4_ system at 250 °C released ∼5.8 wt% of hydrogen in 60 minutes. The addition of NiCo_2_O_4_ significantly enhanced the dehydrogenation kinetics of the surface oxidized LiBH_4_ system. Moreover, the estimated hydrogen binding energy of the LiBH_4_/NiCo_2_O_4_ systems lies in the range of 0.26–0.28 eV. Therefore, it can be inferred that the interaction between H_2_ molecules and LiBH_4_/NiCo_2_O_4_ systems is weak chemisorption or strong physisorption. The remarkable hydrogen storage performance was shown by the LiBH_4_ + 75% NiCo_2_O_4_ system possibly due to the larger surface area, porosity and favourable electronic environment of NiCo_2_O_4_. Moreover, these results provide new opportunities to consider LiBH_4_/NiCo_2_O_4_ systems as an economical and prospective material for hydrogen charging/discharging applications.

## Data availability

The data supporting this article are included in ESI.†

## Author contributions

Ajaijawahar Kaliyaperumal: conceptualization, methodology, data curation, formal analysis, writing – original draft, writing – review & editing. Gokuladeepan Periyasamy: formal analysis, validation, writing – review & editing. Iyakutti Kombiah: formal analysis, validation, investigation, writing – review & editing. Karthigeyan Annamalai: conceptualization, resources, investigation, supervision, writing – original draft, writing – review & editing.

## Conflicts of interest

There are no conflicts of interest to declare.

## Supplementary Material

RA-014-D4RA01709A-s001
