# Supplementary material for: Effect of a mesoporous NiCo2O4 urchin-like structure catalyzed with a surface oxidized LiBH4 system for reversible hydrogen storage applications
Source: RSC Adv. 2024 Jul 2;14(29):20867–78. doi: 10.1039/d4ra01709a (PMC11217879; doi:10.1039/d4ra01709a)
Supplement: RA-014-D4RA01709A-s001 [file RA-014-D4RA01709A-s001.pdf]

Electronic Supplementary Material (ESI) for RSC Advances.
This journal is © The Royal Society of Chemistry 2024

Electronic Supplementary Information

Effect of mesoporous $NiCo_2O_4$ urchin-like structure catalyzed with surface oxidized $LiBH_4$ system for reversible hydrogen storage applications

Ajaijawahar Kaliyaperumal, Gokuladeepan Periyasamy, Iyakutti Kombiah, Karthigeyan Annamalai*

Hydrogen Storage Materials and Nanosensors Laboratory, Department of Physics and Nanotechnology, College of Engineering and Technology, SRM Institute of Science and Technology, Kattankulathur, Chengalpattu, Tamil Nadu, India. 603203

*Corresponding author e-mail id: karthiga@srmist.edu.in

Corresponding Author Address:

Dr. A. Karthigeyan

Professor and Head In-Charge

Department of Physics and Nanotechnology

SRM Institute of Science and Technology

Kattankulathur, Chengalpattu, Tamilnadu, India-603 203

Phone: +91 9841615368

Email: karthiga@srmist.edu.in

Characterization techniques

The Powder X-ray diffraction (PXRD) characterization was performed by PANanalytical X'pert pro diffractometer (Cu Kα = 1.54059 Å, PANanalytical X-Pert Pro, Netherlands) to confirm phase structures of the samples. Fourier transform infrared spectroscopy characterization was examined by SHIMADZU, FTIR spectrometer through ATR mode from 400 to 4000 cm^{-1} to identify the presence of functional groups. BET analyzer (Quantachrome Instruments, Autosorb IQ series) was used to investigate the specific surface area, pore size distribution and average pore volume of the $NiCo_2O_4$ and $LiBH_4$+75% $NiCo_2O_4$ samples. The high resolution scanning electron microscopy and EDS spectra were collected by Thermoscientific Apreo S instrument. Transmission electron microscopy with corresponding SAED patterns were obtained from JEOL Japan, JEM-2100 Plus instrument. The X-ray photoelectron spectroscopy (XPS) was performed by Thermo Scientific MULTILAB 2000 (with monochromatic Al Kα as the X-ray source) instrument to characterize the binding energies and chemical compositions of the samples. Moreover, the thermal stability and thermal properties of the samples were performed with help of thermal analyzer (TG-STA-7200, Hitachi, Japan) from RT to 275 °C with 15 °C min^{-1} rate under argon atmosphere with 100 mL min^{-1} gas flow.

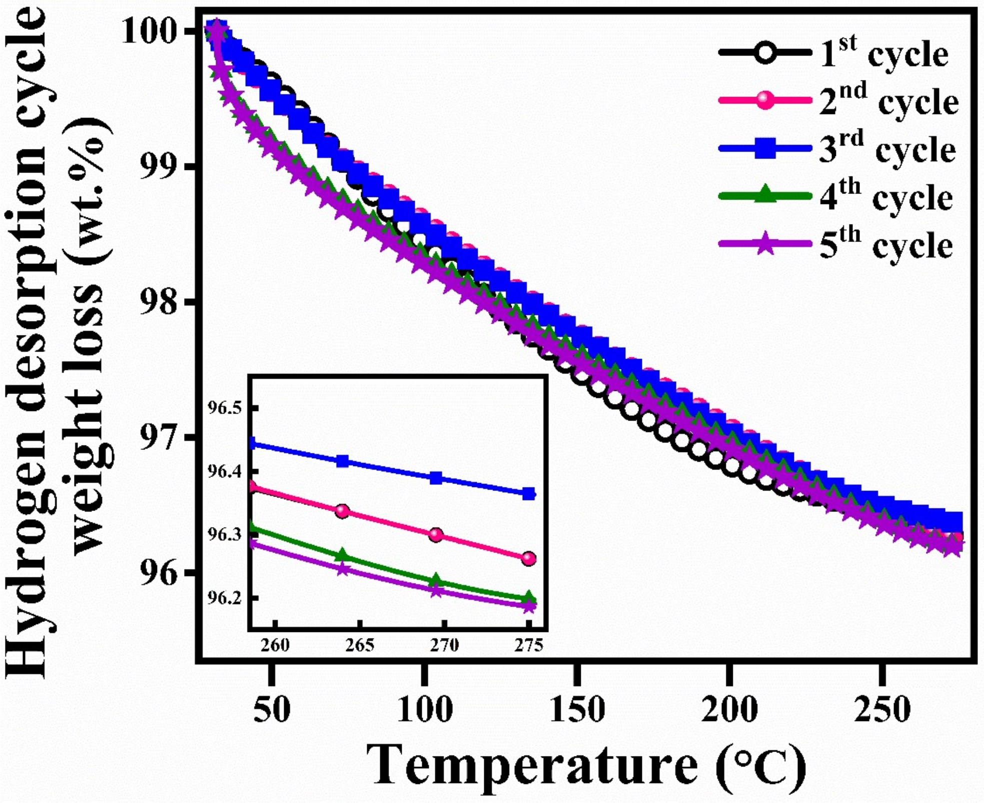

Figure S1. H_2 sorption and desorption cycles of $LiBH_4$+75% $NiCo_2O_4$ system

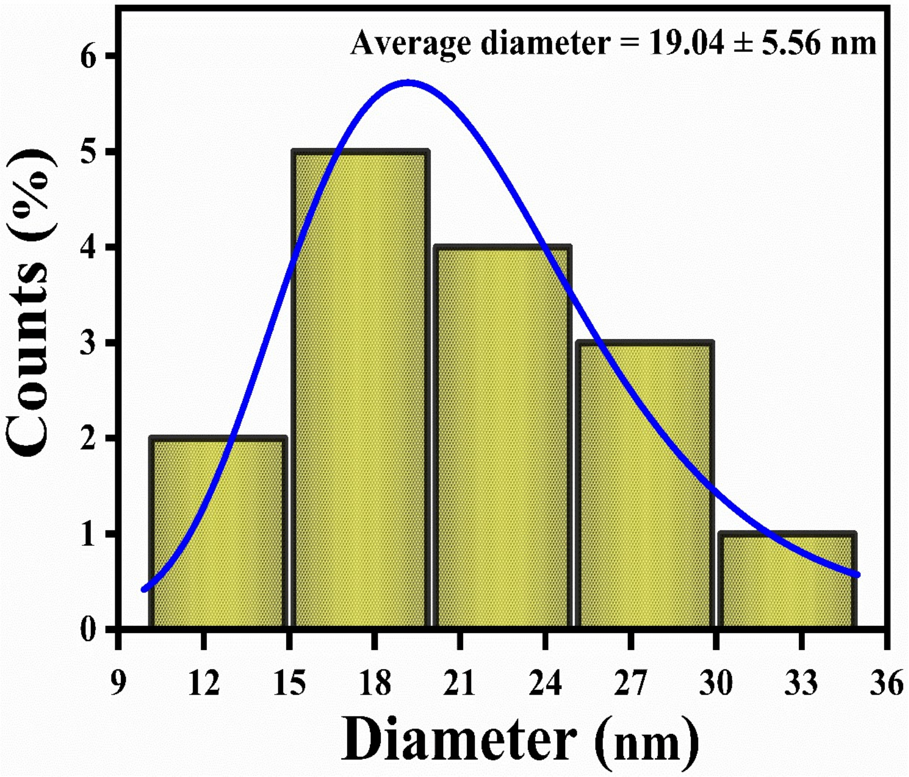

Figure S2. Grain size distribution of $LiBH_4$+75% $NiCo_2O_4$ system

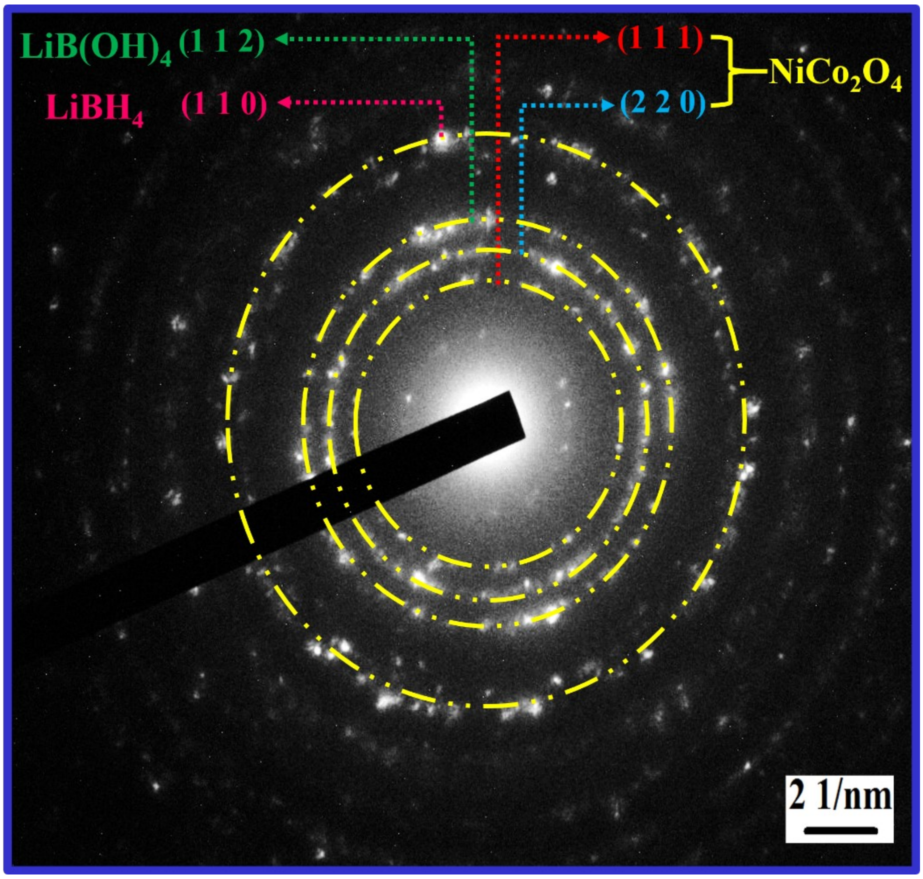

Figure S3. SAED pattern of $LiBH_4$+75% $NiCo_2O_4$ system

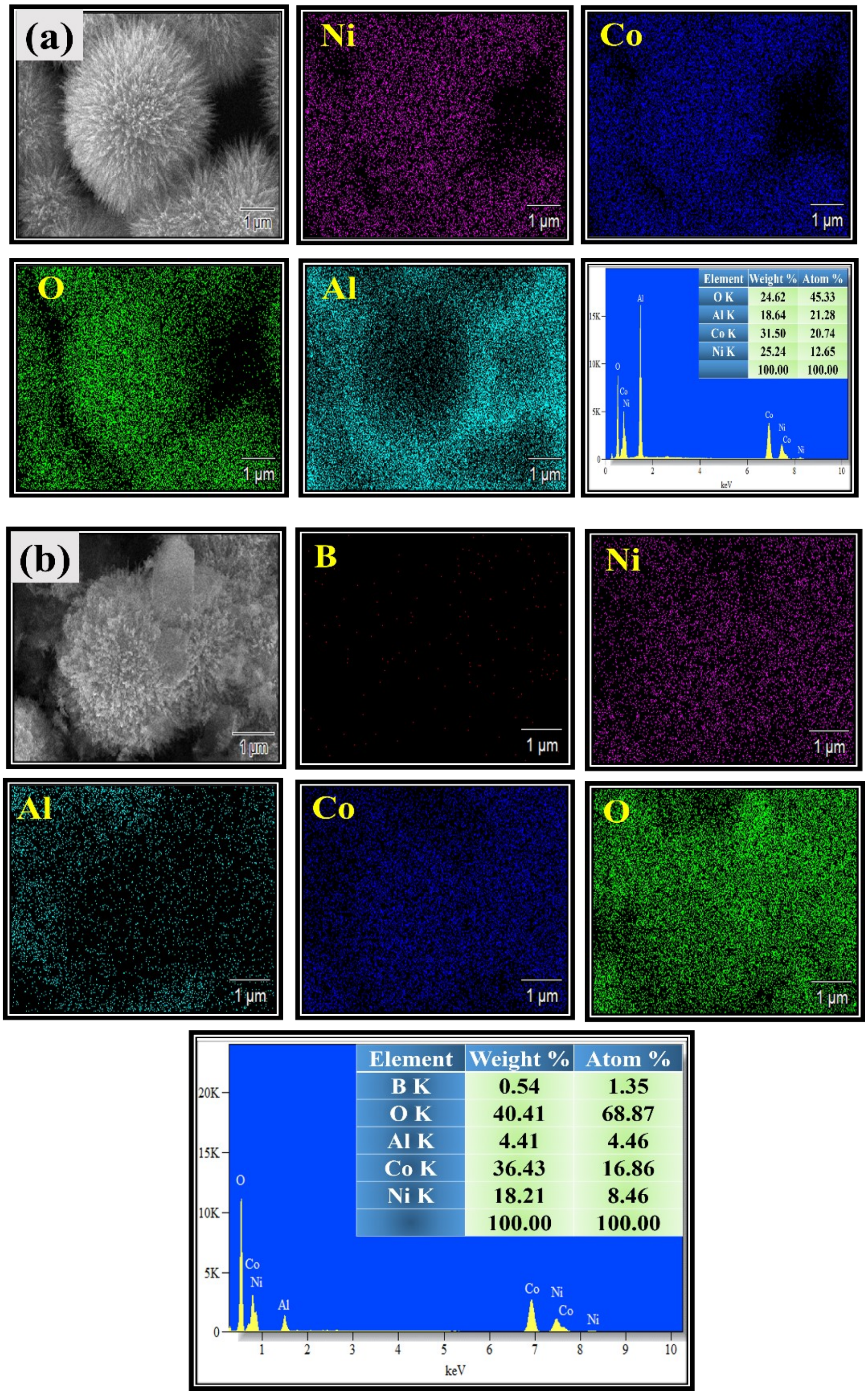

Figure S4. Elemental mapping and EDS profiles of (a) $NiCo_2O_4$ and (b) $LiBH_4$+75% $NiCo_2O_4$ system

Figure S4 displays the EDS and elemental mapping profiles of $NiCo_2O_4$ and $LiBH_4$+75% $NiCo_2O_4$ samples. **Figure S4a** shows the elemental composition of $NiCo_2O_4$, the

urchin-like structure showed uniform distribution of Nickel, Cobalt and Oxygen elements. EDS profile confirms the presence of the Nickel, Cobalt and Oxygen with atomic ratio of 12.65, 20.74 and 45.33 wt.%, respectively. As shown in **Figure S4b**, the $LiBH_4$+75% $NiCo_2O_4$ system consists of Boron, Nickel, Cobalt and Oxygen with atomic ratio of 1.35, 8.46, 16.86 and 68.87 wt.%, respectively. The elemental mapping profiles depicts the existence of Boron, Nickel, Cobalt and Oxygen elements. As shown in the above results, Al content was detected due to sample substrates.
